# Clinical response to discontinuation of anti-TNF therapy in patients with ankylosing spondylitis after 3 years of continuous treatment with infliximab

**DOI:** 10.1186/ar1693

**Published:** 2005-02-21

**Authors:** Xenofon Baraliakos, Joachim Listing, Jan Brandt, Martin Rudwaleit, Joachim Sieper, Juergen Braun

**Affiliations:** 1 Rheumazentrum Ruhrgebiet, Herne, Germany; 2 German Rheumatism Research Center, Berlin, Germany; 3 Charité, Medical University of Berlin, Campus Benjamin Franklin, Department of Rheumatology, Germany

## Abstract

We analyzed the clinical response and the time to relapse after discontinuation of continuous long-term infliximab therapy in patients with ankylosing spondylitis (AS). After 3 years of infliximab therapy, all AS patients (*n *= 42) discontinued treatment (time point (TP)1) and were visited regularly for 1 year in order to assess the time to relapse (TP2). Relapse was defined as an increase to a value ≥ 4 on the Bath Ankylosing Spondylitis Disease Activity Index (BASDAI) and a physician's global assessment ≥ 4 according to the recommendations of the Assessments in Ankylosing Spondylitis (ASAS) working group. After 52 weeks, 41 of the 42 patients (97.6%) had to be reinfused because of relapse. The mean change in the BASDAI between TP1 and TP2 was 3.6 ± 1.7 and that in the physician's global assessment was 4.4 ± 1.8 (both *P *< 0.001). The mean time to relapse was 17.5 weeks (± 7.9 weeks, range 7 to 45). Ten patients (24%) showed a relapse within 12 weeks and 38 patients (90.5%), within 36 weeks. After 52 weeks, only one patient had remained in ongoing remission without further treatment with anti-tumor-necrosis factor. Patients who were in partial remission according to the ASAS criteria and those with normal C-reactive protein levels at the time point of withdrawal had longer times to relapse after discontinuation of the treatment. Retreatment with infliximab was safe and resulted in clinical improvement in all patients to a state similar to that before the treatment was stopped. Discontinuation of long-term therapy with infliximab eventually led to relapse of disease activity in all patients but one.

## Introduction

Ankylosing spondylitis (AS) is a chronic, immune-mediated inflammatory disease that is associated with inflammation in the sacroiliac joints, the axial skeleton, entheses, peripheral joints, the uvea, and other structures [1-3]. In randomized clinical trials, agents targeting the proinflammatory cytokine tumor necrosis factor (TNF)-α, such as the monoclonal antibody infliximab, have produced significant improvement of signs and symptoms in AS patients [4]. Persistence of clinical response was reported in long-term follow-up studies over 2 [5] and 3 years [6]. These results have been substantiated in studies using magnetic resonance imaging of the spine [7].

We reasoned that it was unclear whether after 3 years of successful therapy with infliximab our patients still needed treatment. Similarly, it was unknown whether discontinuation of the infliximab would be tolerated and whether a restart would be efficacious and safe. Furthermore, nothing was known about the clinical parameters predictive of flare after discontinuation of infliximab therapy. Therefore, we decided to study these questions in our cohort, who had been treated with infliximab for the preceding 3 years [6].

## Materials and methods

### Patients and study protocol

The AS patients included in this study had all been receiving infliximab for the preceding 3 years, having participated in the first published randomized clinical trial on this therapy in active AS [4,5,8,9]. After the initial, placebo-controlled phase of that trial, the patients entered open extension phases, in which they were treated continuously with 5 mg/kg infliximab every 6 weeks. At the end of the third year of the study (defined as time point (TP)1), all the patients (*n *= 43) had the opportunity to continue for another extension phase. Only one patient discontinued, because of a side effect. All the others (*n *= 42) were included in the present extension. In accordance with the study protocol, they gave their informed consent and agreed to discontinuation of the infliximab treatment.

The study was approved by the local ethics committee of each site that participated in this multicenter trial.

Thereafter they were visited regularly at 6-week intervals for assessment of their clinical disease state and the time to relapse (TtR). Relapse was defined as a Bath Ankylosing Spondylitis Disease Activity Index (BASDAI) value ≥ 4 [10]*and *a physician's global assessment score ≥ 4 according to the recommendations of the Assessments in Ankylosing Spondylitis (ASAS) working group [11]. Patients were invited to present to the centers between the 6-week intervals at any time if symptoms suggestive of relapse or other problems occurred, and if they did, their clinical symptoms were documented accordingly. In cases of relapse, the patients were reinfused with infliximab at 5 mg/kg (TP2) and were then followed up for 12 weeks after the first reinfusion. All the patients were offered an opportunity to enter the next phase of the trial, for another 2 years.

### Assessment of the individual disease course after discontinuation

Clinical data were assessed at TP1 and TP2 by use of the standard indicators: disease activity as measured by the BASDAI, C-reactive protein (CRP), and erythrocyte sedimentation rate (ESR). Function was assessed according to the Bath Ankylosing Spondylitis Functional Index (BASFI) [12], and mobility was assessed according to the Bath AS Metrology Index (BASMI) [13]. The patient's global assessment score, the physician's global assessment score, and the numerical rating scale for pain (NRS-P) were each assessed on a numerical rating scale ranging from 0 to 10.

### Statistical analysis

The correlation of the data at the two time points was calculated using Pearson's correlation coefficient. The clinical and laboratory data for the patients who experienced a relapse (that is, at TP2) were compared with the data found at TP1.

A Kaplan-Meier survival analysis was used to calculate the probability of a relapse, with duration of response as survival time and relapse as a binomial covariate for the end point. A Cox proportional hazards regression analysis was used to identify possible predictors of flare.

In addition, patients were stratified both according to their BASDAI values at the time of discontinuation, using a cutoff value of 3 at TP1, and also according to the ASAS working group criteria for partial remission at TP1 [14]. Partial remission was defined as a score ≤ 2 (on a scale of 0 to 10) in each of the four ASAS 20% domains, according to the ASAS criteria. The TtR in these groups was compared using a log-rank test. All statistical tests were two-tailed.

## Results

### Baseline findings, at discontinuation of anti-TNF therapy

Table 1 summarizes the mean ages of the patients, their scores on the various measures of AS, the mean ESR, and the mean CRP concentration at TP1, when anti-TNF treatment was discontinued.

The BASDAI values at TP1 were >3 for 13 (31%) of the 42 patients and >4 for 8 (19%) of the 42. The latter were still receiving treatment, because they had experienced a significant decrease of their BASDAI values, of about 30% compared with their baseline value at the start of the study (mean BASDAI 7.4 at baseline versus 5.3 at TP1) and reported definite subjective improvement. At TP 1, 14 (33%) of the 42 patients were in partial remission [14].

### Duration of response after discontinuation

**Figure 1:**
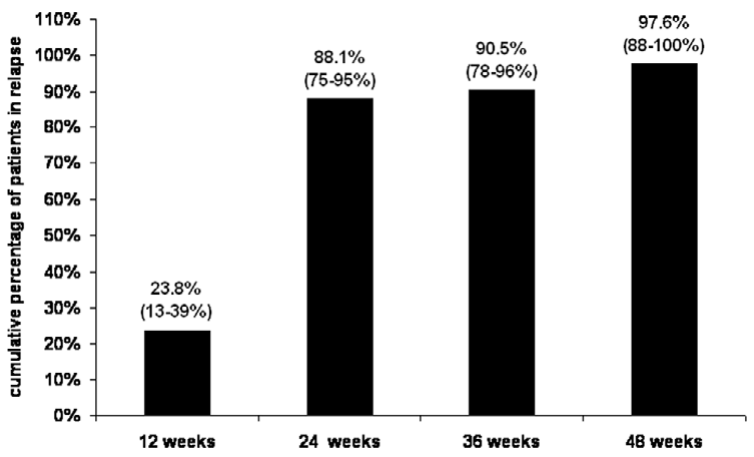
Cumulative percentages (confidence intervals) of retreatment after discontinuation of infliximab in patients treated for ankylosing spondylitis. Retreatment depended on the duration of response to the initial treatment. Of the 42 patients, 10 had to be retreated within 12 weeks after discontinuation of infliximab infusions, 37 within 24 weeks, and 38 within 36 weeks. By week 48, 1 of the 42 patients had not needed retreatment and 41 were again receiving infliximab.

By 3 weeks after the last patient reached TP2, 41 of the 42 patients were being reinfused because of relapse (Fig. 1). Although the first patient reached TP2 after 7 weeks, it took the last patient more than 52 weeks. However, most patients (64%) experienced a flare between week 12 (10/42 patients; 23.8%) and week 24 (37/42 patients; 88.1%). The mean time between TP 1 and TP 2 was 17.5 weeks (± 7.9 weeks, range 7 to 45) and the median time was 15 weeks.

### Means and changes of the assessed parameters after discontinuation of treatment

Between TP1 and TP2, the mean increase of the BASDAI was 3.6 (± 1.7), the mean increase of CRP was 17.6 mg/l (± 23.4 mg/l), and the mean increase of the ESR was 21.0 mm/hour (± 29.7 mm/hour), (all *P *< 0.001 in comparison of TP1 with TP2). All changes between the two time points were statistically significant (Table 1).

### Correlations between the individual parameters

The changes in the BASDAI correlated well with the changes in the BASMI (*r *= 0.35, *P *= 0.03) and the BASFI (*r *= 0.79, *P *< 0.001). The changes in these three indexes correlated well with the changes in the patient's global assessment score (*r *= 0.81, *r *= 0.32, and *r *= 0.74, respectively; all *P *< 0.05) and in the physician's global assessment score (*r *= 0.49, *r *= 0.39, and *r *= 0.46, respectively; all *P *< 0.05). The change in the NRS-P correlated well with the change in all clinical findings but not with the laboratory values (data not shown). The TtR was not correlated with any clinical parameter.

### Correlations between clinical remission and disease activity and response to discontinuation of treatment

**Figure 2:**
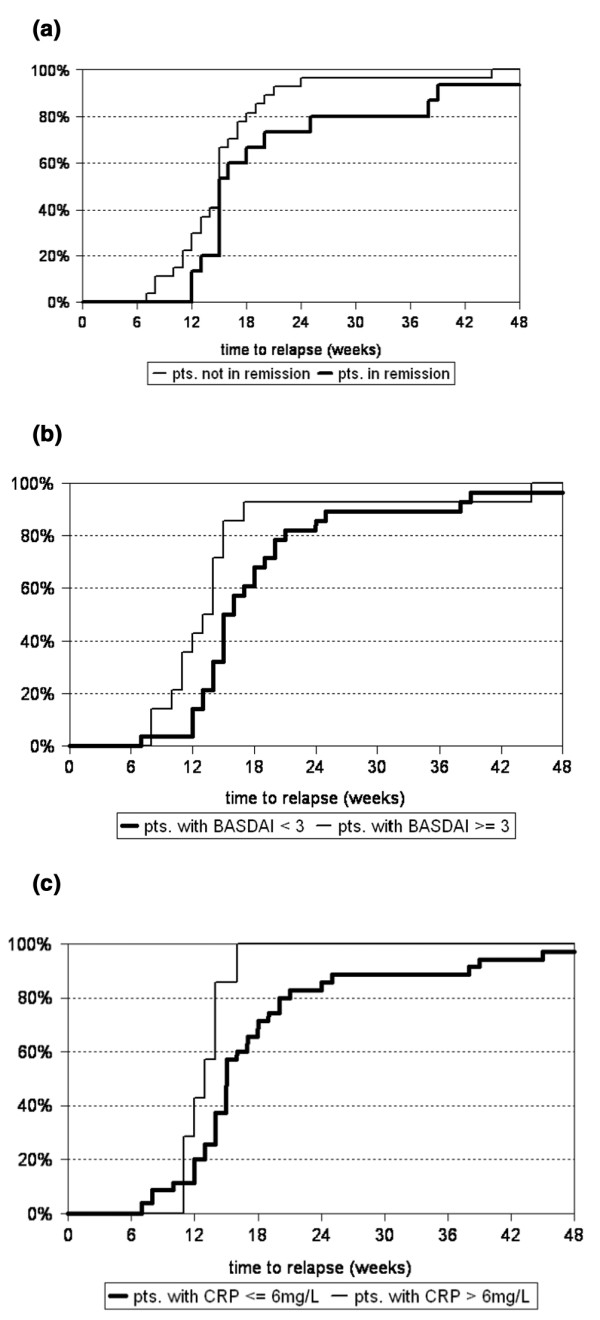
Kaplan-Meier analysis of time to relapse in AS patients after discontinuation of infliximab treatment. **(a) **Cumulative probability of relapse analyzed according to state of remission as measured by ASAS partial remission criteria at TP1. Patients were (bold line) or were not (thin line) in partial remission at TP1. **(b) **Cumulative probability of relapse according to state of disease activity at TP1 as indicated by a BASDAI ≥ 3 (high disease activity) (thin line) or <3 (low disease activity) (bold line). **(c) **Cumulative probability of relapse according to state of disease activity at TP1 as indicated by a CRP ≤ 6 mg/l (bold line; low disease activity) or >6 mg/l (thin line; increased disease activity). AS, ankylosing spondylitis; ASAS, Assessments in Ankylosing Spondylitis [working group]; BASDAI, Bath Ankylosing Spondylitis Disease Activity Index; CRP, C-reactive protein; pts., patients; TP1, time point 1 (when infliximab treatment was discontinued).

Patients in partial remission at TP1 (*n *= 15) had a longer duration of response than patients who did not fulfill remission criteria (*P *= 0.059). The mean TtR was 21.3 weeks (95% confidence interval (CI), 15.5 to 27.2 weeks) for patients in remission but only 15.4 weeks (12.7 to 18.1) for the other group (Fig. 2a).

Similarly, in the analysis of the disease status at TP1, there was also a difference between the patients with low (BASDAI <3) and high (BASDAI ≥ 3) disease activity (Fig. 2b; *P *= 0.039); the mean TtR of the patients with high disease activity was 14.8 weeks (CI 10.0 to 19.6) and the mean TtR of the patients with low disease activity was 18.9 (CI 15.4 to 22.4). This result was confirmed by a Cox regression analysis. A higher BASDAI, an elevated CRP, older age, and a longer disease duration were associated with a shorter TtR. Three of seven patients with a CRP >6 mg/l at the end of year 3 (TP1) had already experienced a relapse by 12 weeks, and the remaining four patients, by 16 weeks (Fig. 2c; *P *= 0.009). The cumulative probability of relapse was less in patients with low CRP levels (20% by week 12 and 60% by week 16, respectively) than in patients with elevated CRP levels (43% by week 12 and 100% by week 16, respectively).

### Response to retreatment

All 41 patients who were reinfused responded well to the restart of therapy with infliximab. They showed a clear improvement of signs and symptoms and reached a disease state similar to that before the treatment was discontinued. The main inclusion and outcome parameter, the BASDAI, had improved from 6.1 ± 1.4 at TP2 to 3.2 ± 2.6 by 6 weeks after reinfusion and to 2.9 ± 2.1 by 12 weeks after reinfusion, respectively (both *P *< 0.001). All other parameters improved similarly well in comparison with TP2 (not shown).

There was no adverse event and no other safety concern after resumption of infliximab therapy.

## Discussion

Infliximab has proven clinical efficacy in patients with active AS, which is associated with definite improvement of disease activity in both the short and the long term, for up to 3 years [5,6]. Our study is the first to examine the clinical response to discontinuation of long-term infliximab therapy in patients with AS. Several important observations were made.

First, we found that discontinuation of long-term therapy with infliximab in patients with AS leads to a clinical relapse of the disease, with deterioration of signs and symptoms, after several weeks to months. This indicates that the majority of patients may, rather, need continuous anti-TNF therapy.

Another finding is that even though there were relapses eventually, in many patients the low disease activity at discontinuation of therapy persisted for some weeks after discontinuation, although only one patient was in ongoing remission for more than 1 year. The mean duration of ongoing response was almost 4 months. Since the time of persistent clinical efficacy of infliximab after discontinuation varied widely between patients, the optimal dose and the optimal infusion interval for infliximab is also likely to be different from patient to patient. The best dosage probably needs to be defined individually.

We also found that there seem to be predictive factors for the duration of clinical improvement after discontinuation of infliximab therapy in AS patients. The data suggest that clinical improvement persists longer when a state of partial remission, low disease activity, and low CRP levels are present at the time of discontinuation. Thus, the outcome after discontinuation can be partly predicted.

These conclusions are complementary to those predictive of major response that have been reported recently [15]. Overall, it seems that patients who may be candidates for discontinuation or a possible extension of infusion intervals of infliximab therapy have a better outcome if this decision is made while the patients are in a state of low disease activity. Such patients are more likely to have ongoing benefit from previous therapy for several more months.

The favorable response after retreatment argues against an important role of formation of antibodies to infliximab (ATI) in these patients. This response is probably due to the preselection of the patients by the previous 3 years of persistent high-dose therapy with infliximab, which clearly differs from other approaches [16].

Discontinuation of infliximab may become necessary in various patients: those who are in remission for long periods and simply want to test the remission; those who want to become pregnant and wish to exclude the risk of medication toxicity (although there is no indication that infliximab may be harmful); those with more severe or repetitive infection(s); and those who have to undergo surgery (although there is no reason to think that ongoing infliximab therapy may be harmful, good data are lacking).

Another finding of our study is that discontinuation of infliximab therapy seems justified, since we found that retreatment with infliximab was safe, resulting in a good clinical response, similar to that before discontinuation. There was no loss of efficacy and no need for an increased dose after the new start of infliximab therapy. Thus, if for any reason discontinuation of anti-TNF therapy is considered necessary, that seems possible with no major problems regarding efficacy and safety. This may have definite implications for daily practice, since discontinuation of therapy at certain intervals, such as after 1 or 2 years of therapy, may become a standard approach. Payers and patients may want to make sure that further anti-TNF therapy is needed. An intermittent cessation of anti-TNF therapy may be considered in the case of patients who respond well to infliximab therapy for longer periods of time. Since it is unknown how long the patients should receive anti-TNF therapy, it is unclear how to deal with this uncertainty in clinical practice. One possible approach would be to check from time to time whether the disease is still active or has become active again after initial improvement due to infliximab therapy. Another possibility would be to slowly extend the intervals between infusions. This approach would obviously have important economic implications.

However, we think that no clear recommendation for such an approach can be given in the light of present knowledge. More work is needed to confirm our findings and further studies are required to better clarify these issues.

The decision to use a BASDAI cutoff score of 4 is based on the ASAS recommendations. The decision to use a cutoff score of 3 to indicate low disease activity is, at the moment, arbitrary but may serve as a basis for further discussion. It will be especially interesting to learn from the patients whether a score of 3 comes closer to indicating an acceptable state.

## Conclusion

Therapy with infliximab has definite long-term clinical efficacy and safety in patients with AS. Patients who discontinue therapy are likely to have a clinical relapse within several weeks to months. Therefore, continuous therapy seems to be necessary for most patients with AS. Importantly, however, we found that retreatment is safe and the clinical efficacy is as good as that before discontinuation. Patients in partial remission or with low disease activity have a longer duration of response after discontinuation than patients with higher disease activity. Overall, anti-TNF therapy is a major step forward in the treatment of patients with AS.

## Competing Interests

Dr Braun and Dr Sieper have received reimbursements and fees from the Centocor Amgen, and Wyeth and Abbott.

## Abbreviations

AS = ankylosing spondylitis; ASAS = Assessments in Ankylosing Spondylitis [working group]; BASDAI = Bath Ankylosing Spondylitis Disease Activity Index; BASFI = Bath Ankylosing Spondylitis Function Index; BASMI = Bath AS Metrology Index; CI = confidence interval; CRP = C-reactive protein; ESR = erythrocyte sedimentation rate; NRS-P = numerical rating scale for pain; TNF = tumor necrosis factor; TP = time point; TtR = time to relapse.

## Authors' contributions

XB: Preparation of data analysis, preparation of the manuscript, study coordination. JL: Data analysis, statistical evaluation. JB: Monitoring and investigation of the patients, study coordination. MR: Monitoring and investigation of the patients, study coordination. JS: Investigator, writing of the manuscript. JB: Idea, writing of the manuscript, principal investigator, responsible for the study. All authors read and approved the final manuscript.

## Figures and Tables

**Table 1:** Clinical and laboratory findings for 42 patients with ankylosing spondylitis treated with infliximab

Finding	BASDAI	BASMI	BASFI	PatGA	PhysGA	NRS-P	ESR (mm/h)	CRP (mg/l)
At time point 1^a^								

Mean ± SD	2.5 ± 1.8**	2.7 ± 2.0*	2.9 ± 2.4**	2.6 ± 1.5**	2.6 ± 2.1**	2.6 ± 2.1**	10.5 ± 7.3**	3.1 ± 4.2**
Median	2.4	2.0	2.5	4.0	2.0	2.0	8.0	1.1
Range	0.0 - 6.8	0.0 - 7.0	0.0 - 8.3	0.0 - 8.0	0.0 - 4.0	0.0 - 7.0	2.0 - 32.0	0.0 - 19.0

At time point 2^a^								

Mean ± SD	6.1 ± 1.4**	3.2 ± 2.2*	5.8 ± 1.8**	6.9 ± 2.1**	7.0 ± 1.5**	7.1 ± 1.7**	31.5 ± 29.7**	20.7 ± 23.7**
Median	6.2	3.0	5.7	7.0	7.0	7.0	23.0	14.0
Range	4.0 - 9.2	0.0 - 9.0	1.2 - 9.1	4.0 - 10.0	4.0 - 10.0	0.0 - 10.0	4.0 - 150.0	0.3 - 126.0

Change between time points 1 and 2								

Mean ± SD	3.6 ± 1.7	0.5 ± 1.5	2.9 ± 2.0	4.3 ± 1.9	4.4 ± 1.8	4.5 ± 2.2	21.0 ± 29.7	17.6 ± 23.4
Median	3.6	0.5	2.5	4.0	4.0	4.0	12.0	11.5
Range	-1.2 - 6.7	-4.0 - 3.0	-0.5 - 7.8	-2.0 - 8.0	-2.0 - 8.0	-1.0 - 8.0	-6.0 - 146.0	-6.3 - 123.0

^a^Time point 1 is the time point at which infliximab treatment was discontinued; time point 2 is that when retreatment began. *P < 0.05, **P < 0.001, when means at time points 1 and 2 are compared. BASDAI, Bath Ankylosing Spondylitis Disease Activity Index; BASFI, Bath Ankylosing Spondylitis Function Index; BASMI, Bath Ankylosing Spondylitis Metrology Index; CRP, C-reactive protein; ESR, erythrocyte sedimentation rate; NRS-P, numerical rating scale for pain; PatGA, patient's global assessment; PhysGA, physician's global assessment; SD, standard deviation.
